# A Review of Exhaled Volatile Organic Compounds as Emerging Diagnostic Tools for Gastrointestinal Malignancies

**DOI:** 10.34172/aim.34980

**Published:** 2026-01-01

**Authors:** Amirreza Ghafourian, Masoomeh Hamdi, Maryam Rayatpisheh, Ramin Shakeri, Naser Ahmadbeigi, Shokoofeh Borzabadi, Abolghasem Jouyban, Reza Ghanbari

**Affiliations:** ^1^Department of Medical Laboratory Sciences, School of Allied Medical Sciences, Tehran University of Medical Sciences, Tehran, Iran; ^2^Digestive Oncology Research Center, Digestive Diseases Research Institute, Shariati Hospital, Tehran University of Medical Sciences, Tehran, Iran; ^3^Gene Therapy Research Center, Digestive Diseases Research Institute, Shariati Hospital, Tehran University of Medical Sciences, Tehran, Iran; ^4^Department of Biology, Science and Research Branch, Islamic Azad University, Tehran, Iran; ^5^Academy of Medical Sciences IR, Iran, Tehran, Iran; ^6^Pharmaceutical Analysis Research Center, Pharmaceutical Sciences Institute, Tabriz University of Medical Sciences, Tabriz, Iran

**Keywords:** Cancer biomarkers, Exhaled breath, Gastrointestinal cancers, Non-invasive diagnostics, Volatile organic compounds

## Abstract

Gastrointestinal cancers encompass a range of malignancies, including esophageal, gastric, esophagogastric, hepatocellular, pancreatic, and intestinal cancers. Considering the prevalence of these cancers, alongside their fatal nature, and the nonspecific symptoms that often arise in the advanced stages, developing a non-invasive screening/diagnostic method capable of detecting cancer early is in high demand. Exhaled breath serves as an accessible source of biomarkers, including volatile organic compounds (VOCs), a promising type of diagnostic marker. This method requires a sample as small as five breaths. Exhaled breath analysis is conducted using different mass spectrometry methods coupled with nano sensors or neural networks. Furthermore, innovative devices like electronic noses have also shown great promise in diagnosing different types of cancers. These biomarkers show potential in reliably differentiating between healthy individuals and cancer patients. Additionally, these compounds can be utilized for cancer staging and monitoring treatment response. However, certain fallbacks persist in differentiating malignant from pre-malignant conditions. Despite the promise of this diagnostic approach, certain limitations, such as variations in VOC profiles across different populations, lack of standardized collection protocols, and absence of established universal reference standards, underscore the need for proper standardization and large-scale, multi-center validation studies.

## Introduction

 Gastrointestinal (GI) cancers encompass a wide range of malignancies, including cancers of the esophagus, stomach, liver, pancreas, and intestines. Representing over 26% of the global cancer incidence and over 35% of deaths from cancer in 2018, GI cancers remain a major global health issue.^1^ Esophageal and gastric cancers are predominantly observed in developing countries, whereas colorectal cancer (CRC) is the most prevalent GI cancer in developed nations.^2^

 Despite the availability of various cancer diagnostic methods, histological biopsy performed under endoscopy remains the most common approach for diagnosing gastrointestinal cancer. However, its invasive nature, along with significant economic burden and associated discomfort, has prompted the need for development of new diagnostic techniques.^3^ Advancement of these new methods relies on identification of novel biomarkers. Although there are several predictive and prognostic biomarkers available, the high mortality rates in patients with GI cancer underscore the critical need to enhance innovative diagnostic biomarker strategies for early detection of these malignancies.^4^

 Exhaled breath (EB) presents a promising matrix for the discovery of biomarkers. This sample contains a diverse array of molecules, including both volatile and small-molecule compounds. This completely non-invasive method requires as few as five breaths collected in inert bags such as Mylar, Tedlar, Nalophan, and steel breath bags, or even cans. The amount utilized as an adequate sample for the analysis of multiple biomarkers is between 20 mL and 4 L.^3,5^ The concentration of volatile compounds such as acetic acid, formic acid, and ammonia is significantly higher than non-volatile constituents in EB. Among these, volatile organic compounds (VOCs) offer valuable insight into an individual’s health status.^5,6^

 A variety of methodologies have been employed to examine VOCs to identify potential cancer biomarkers. Gas chromatography coupled with mass spectrometry (GC-MS) has been widely utilized for detection of VOCs associated with cancer.^7^ GC-MS works through combining gas chromatography’s separation power with mass spectrometry’s identification capabilities. In this process, a sample is volatilized and carried through a capillary column where components separate based on volatility and interactions. Each eluting compound is then ionized, fragmented, and analyzed by a mass spectrometer to generate a characteristic mass spectrum for identification and quantitation.^8^ GC–MS is widely recognized as a versatile analytical platform with high robustness, excellent separation capabilities, selectivity, sensitivity, and reproducibility, as well as user-friendliness in terms of analysis time and operating costs, as well as its capability to facilitate compound identification.^9^ Beyond classical GC-MS, more innovative GC-MS methods, including thermal desorption (TD) GC-MS^10-12^ and solid-phase microextraction (SPME)-GC/MS,^13-17^ have been also utilized. These methods are coupled with nano-sensors or probabilistic neural network (PNN) numeric analysis tools to collect more data. Moreover, using flow tube mass spectrometry (SIFT-MS) is also a quite common method. Additionally, one study used self-made proton transfer reaction mass spectrometry (PTR-MS),^10^ whilst another one used ion molecule reaction mass spectrometry (IRM-MS).^11^ Although GC-MS is widely used, it does have certain limitations that have led to an increased interest in electronic noses (e-nose). These innovative devices offer the ability for simultaneous achievement of selectivity and specificity of multiple VOCs through the use of an array of sensors.^12^

 VOCs originate from both internal and external sources. Endogenous markers, which are produced from metabolic processes within the body, include hydrocarbons, and substances containing oxygen, sulfur, carbon disulfide, or nitrogen. These markers are frequently utilized for diagnostic purposes. These compounds are found in approximate concentrations of 10^-12^ mol/L to 10^-9^ mol/L in physiological conditions, while elevated levels can be observed in states of inflammation and various diseases.^13^ Recently, research on the correlation of these metabolites and malignancies has been highlighted.

 Recent studies demonstrate significantly different levels of VOCs in patients with various types of cancer, including breast cancer,^14^ oral squamous cell carcinoma (OSCC) patients,^15^ and head and neck squamous cell carcinoma (HNSCC), as well as lung, prostate, gastric, colorectal, and liver cancers.^16^ Yet, there is a critical need for standardized study protocols, validation across diverse populations, and a deeper understanding of VOC biomarkers along with a comprehensive overview of various related studies. Therefore, we aim to review the use of EB and its components, particularly VOCs, in early detection of GI cancers, to provide a comprehensive overview of this innovative method and its potential to influence future diagnostic strategies for these serious and widespread malignancies.

## Colorectal Cancer

 Colorectal cancer (CRC), the third most commonly diagnosed and the second leading cause of cancer death worldwide, is influenced by lifestyle and hereditary factors.^17^ Since symptoms typically manifest only in the later stages of CRC, global screening initiatives are being implemented to promote early diagnosis and to reduce the associated morbidity and mortality rates.^18^ However, the global burden continues to rise, especially in developing nations.^17^ Therefore, finding inexpensive, accessible, and useful screening/diagnostic methods is crucial. Right now, various efforts are underway to identify innovative and effective biomarkers and detection methods for early detection of this fatal and widespread disease. In the context of CRC, studies have suggested that it is feasible to differentiate CRC patients and healthy individuals via analysis of VOCs in EB.

 Initially, 6 VOCs (1,3-dimethyl benzene, 1,1′-(1-butenylidene)bis benzene, 1-iodo nonane, [(1,1-dimethylethyl)thio] acetic acid, 2-amino-5-isopropyl-8-methyl-1-azulenecarbonitrile, and 4-(4-propylcyclohexyl)-4′-cyano[1,1′-biphenyl]-4-yl ester benzoic acid) were identified as potential markers for differentiating patients with colon cancer from healthy individuals.^19^ In a related study, several biomarkers exhibited significant differences in expression among CRC patients, with eight compounds (4-ethyl-1-octyn-3-ol, ethylaniline, cyclooctylmethanol, cyclohexanone, 2,2-dimethyldecane, trans-2-dodecen-1-ol, dodecane, and 3-hydroxy-2,4,4-trimethylpentyl 2-methylpropanoate) showing upregulation and levels of 6-t-butyl-2,2,9,9-tetramethyl-3,5-decadien-7-yne being downregulated in CRC patients compared to the normal population (20). Although most identified compounds belong to similar categories, such as aldehydes, alkanes, ketones, and other volatile substances, the observed differences, as indicated, are likely attributed to various analytical techniques, statistical methodologies, and potential variations in lipid composition and distribution.^20^

 Moreover, indole and benzaldehyde were indicated to be increased, while ethyl benzene was decreased in the case of colorectal cancer.^21^ Also, it was identified that four VOCs, including ethyl-acetate, acetone, ethanol, and 4-methyl octane, have different levels in colorectal patients in comparison to healthy subjects.^22^ Additionally, 15 other VOCs with significant diagnostic performance were identified using PNN with an accuracy higher than 75%.^23^

 In another study, seven compounds were statistically different between cancerous patients and the control group. Among these, propanal (NO + ) exhibited a significant increase in cancer. Applying a threshold of 28 ppbv, CRC could be diagnosed with 96% sensitivity and 76% specificity. Additionally, following surgical resection, propanal levels decreased to those consistent with control patients, but noticeably increased again with CRC recurrence.^3^

 In a study by Altomare *et al.*,^24^ a total of 31 VOCs were expressed at significantly different levels among CRC patients in the Follow Up (FU) group, those in the Follow (F) group, and healthy controls (HC). Using these 31 VOCs, a probabilistic neural network (PNN) analysis was performed. The model showed strong reliability in distinguishing between the CRC and FU groups, achieving 100% sensitivity, 95.83% specificity, 97.50% accuracy, and an area under the curve (AUC) of 0.993. Similarly, when comparing the FU group with the HC group, this VOC dataset yielded 100% sensitivity, 96.36% specificity, 97.70% accuracy, and an AUC of 0.992. Comparing these results with those from a previous study by this research team,^23^ 11 common compounds were found. Using these 11 compounds, CRC patients and FU patients were distinguished, with 100% sensitivity, 97.92% specificity, 98.75% accuracy, alongside an AUC of 1, with only one patient misclassified. Furthermore, comparing the FU and HC group, the same set of 11 VOCs exhibited 100% sensitivity, 90.91% specificity, 94.25% accuracy, and an AUC of 0.959.^23^ This study further indicated a potential correlation between VOCs and cancer screening, as well as secondary prevention strategies.^3^

 Furthermore, breath analysis utilizing the final model of e-nose for diagnosing CRC demonstrated 95% sensitivity and 64% specificity, achieving an AUC of 0.84 in discriminating CRC patients from healthy individuals. The evaluation also included benign conditions such as advanced adenomas or non-advanced adenomas, and hyperplastic polyps. While the e-nose effectively differentiated CRC patients from healthy controls, it struggled to distinguish between CRC and advanced adenomas, as well as between advanced and non-advanced adenomas. This limitation suggests that the VOC profiles associated with these conditions are too similar.^25^ This limitation should be taken into account in further evaluations to increase this method’s reliability.

## Gastric Cancers

 Gastric Cancer (GC) is among the leading causes of mortality globally.^26^ Due to its lethal nature and the absence of distinct clinical symptoms in the early stages, the need for innovative biomarkers for early detection of this cancer is crucial.^27^ To answer this demand, several studies have explored the potential of metabolites found in EB to diagnose GC and also to differentiate it from similar conditions.

 Initially, it was observed that 2-propenenitrile, 2-butoxyethanol, 6-methyl-5-hepten-2-one, furfural, and isoprene were expressed more abundantly in patients with GC and/or peptic ulcers than in those with less severe gastric conditions. Furthermore, using discriminant factor analysis (DFA), predictive models were developed that effectively distinguished between gastric cancer and benign gastric conditions, achieving 89% sensitivity and 90% specificity. Additionally, there were models capable of differentiating patients across various cancer stages and among different benign conditions.^27^ A related study conducted by Amal *et al*. found that cancer patients and those at high risk exhibited distinct respiratory markers. The researchers identified eight VOCs with significantly different expression levels between the groups, including furfural, 2-butyloxyethanol, 2-propenenitrile, hexadecane, 4-methyloctane, α-methyl-styrene, 1,2,3-trimethylbenzene, and 2-butanone. Utilizing a combination of nanoarrays and methods for pattern recognition, the study demonstrated the ability to differentiate between GC patients and healthy individuals (operative link on gastric intestinal metaplasia (OLGIM) 0-IV) with 73% sensitivity and 98% specificity. Notably, the subgroups were effectively distinguished.^28^ These two studies had three common VOCs, including 2-butoxy-ethanol, furfural, and 2-propenenitrile, revealing their great potential for the diagnosis of GC.

 A study conducted on the Colombian population identified six compounds with significantly different expression levels between patients with GC and healthy individuals. These compounds included trans-2, 2-dimethyl-3-decene, hexadecane, octadecane, m-xylene, 1-cyclohexyl-2-(cyclohexylmethyl) pentane, and eicosane. Utilizing principal component analysis (PCA), the classification model achieved an impressive accuracy rate of 97%, along with 100% sensitivity and 93% specificity. It is noteworthy that this study indicated differences between this study and the previous ones with different populations^27,28^ to be related to lifestyle, alimentation, and genetic differences.^29^

 Moreover, 14 VOCs have been identified that can differentiate healthy individuals from those with early GC (EGC) and advanced GC (AGC). These VOCs included, 2-methylpentane, 3-methylpentane, acetone, phenyl acetate, isoprene, hexane, 2-methylhexane, 3-methylhexane, 2,3-dimethylpentane, pivalic acid, tetradecane, hexanol, menthol, and dodecane, containing saturated hydrocarbons which are particularly abundant in the breath of GC patients due to their formation from lipid peroxidation—an effect that contributes to tissue damage associated with cancer.^30,31^ Additionally, a breath analyzing technique utilizing a surface-enhanced Raman scattering (SERS) sensor has been developed as a detector. This sensor distinguished healthy subjects from patients with GC, and also determined whether the cancer is at an early or advanced stage with a specificity of more than 92% and a sensitivity of more than 83%.^31^

 Interestingly, in a study using a back-propagation neural network (BPN), 93% accuracy, 94.38% sensitivity, and 89.93% specificity were achieved in distinguishing normal, suspected, and positive cases of GC.^32^

 Results also indicated that three metabolites, including hexadecane, undecane, 3,8-dimethyl-, and 2,3-butanediol, showed upregulated levels in carcinoma patients compared to normal controls. Also, 1,3-dioxolan-2-one was significantly downregulated in patients with carcinoma than in healthy subjects and/or subjects with gastric ulcers.^33^

 In addition, it was indicated that there are higher relative peak intensities of acetone, 1,3-dioxolan-2-one, isoprene, phenol, 1,2,3-trimethylbenzene, meta-xylene, and phenyl acetate in the breath samples of gastric cancer patients. Using a developed diagnostic prediction model with 121 samples in the training set and 53 samples in the test set, 96.2% accuracy was reached. Also, the AUC of 0.997 demonstrated the model’s excellent predictive capability.^34^

 Furthermore, aiming to explore the capability of an e-nose to differentiate healthy and cancerous histology by EB analysis, the findings indicated a differential ability with 81% sensitivity, 71% specificity, and 75% accuracy. Importantly, these results yield a 62% positive predictive value and an 87% negative predictive value, underscoring the potential of e-nose in diagnosing GC.^35^

 A notable connection has been established between breath VOCs and gut/fecal microbiota. Initial findings indicated that two volatile metabolites, specifically 1-octanol and octane,1,1’-oxybis-, exhibited significantly different levels between cancer patients and control groups. Furthermore, through the application of sparse principal component analysis and canonical correlation analysis (CCA), it was demonstrated that 14 distinct metabolites were identified in the breath of patients with GC, classified as hydrocarbons, alcohols, aromatics, ketones, ethers, and organosulfur compounds, with a significant correlation with 33 fecal bacterial taxa. Also, seven volatile metabolites representing alcohols, aldehydes, esters, phenols, and benzamide derivatives were correlated with 17 bacterial taxa.^36^

 Although these studies have indicated substantial promise in utilizing VOCs in EB for the diagnosis of GC, a multi-center investigation revealed considerable discrepancies in the chemical composition of breath samples collected from study cohorts at two distinct sites. Such variability in sample composition underscores the limitations of diagnostic strategies that depend solely on the quantification of individual VOC concentrations. These findings further emphasize the need for diagnostic methodologies that prioritize the detection of alterations in the overall patterns of disease-specific VOC profiles, rather than focusing exclusively on single-compound measurements.^37^

## Esophageal or Esophago-gastric Cancers

 Due to the aggressive nature, poor prognosis, and high mortality rate associated with esophageal cancer, along with its substantial global impact,^38^ there is a pressing need for applicable methods for screening and early diagnosis of this disease. In this context, various studies have explored the potential of VOCs as accessible and promising biomarkers.

 First, it was indicated that four VOCs, phenol, methyl phenol, ethyl phenol, and hexanoic acid, were found to be differently expressed between esophago-gastric cancer patients and positive controls. Applying ROC analysis for a combination of these VOCs resulted in an AUC of 0.91, highlighting the potential of VOC profiling in identifying patients with esophago-gastric cancer.^39^

 In line with the previous study, a more comprehensive assessment on the VOC profile of gastric and esophageal adenocarcinomas was performed, and 12 VOCs were identified to have elevated levels in both the gastric and esophageal adenocarcinoma groups in comparison to their noncancerous counterparts. The VOCs included pentanoic and hexanoic acid, phenol alongside methyl and ethyl phenol, and also, butanal, pentanal, hexanal, heptanal, octanal, nonanal, and decanal. Additionally, butyric acid was found to be significantly higher in patients with esophageal adenocarcinoma compared to the non-cancerous controls. Notably, there was no significant difference observed in the VOC profiles between esophageal adenocarcinoma and gastric adenocarcinoma. Furthermore, VOCs did not demonstrate any variations in staging within the cancer groups. ROC analysis resulted in an AUC of 0.98 for discriminating GC and 0.97 for differentiating esophageal cancer from the normal upper GI (UGI) tract group. The AUCs for distinguishing patients with gastric and esophageal cancers from non-cancerous individuals (including patients with benign tumors or normal UGI) were 0.92 and 0.90, respectively.^40^

 Furthermore, generating a diagnostic model based on five VOCs, including butyric, pentanoic, and hexanoic acid, as well as butanal and decanal, resulted in an AUC of 0.85, 80% sensitivity, and 81% specificity for the diagnosis of esophagogastric cancer.^41^

 Moreover, using a self-designed PTR-MS, it was revealed that seven ions, including five downregulated ones (m/z 34, m/z 63, m/z 95, m/z 107 and m/z 45) and two upregulated ones (m/z 27, m/z 136), were able to discriminate between the esophageal cancer patients and healthy people with 86.2% sensitivity and 89.5% specificity. Possible substances for these ions were as follows: m/z 63-dimethyl sulfide, thioethanol; m/z 95-phenol, 1, 3-cycloheptadiene, dimethylsulfone, 2-methyl-1, 3-diazine, 1-methylene-2-cyclohexene; m/z 107-ethylbenzene, p-xylene, o-xylene, m-xylene, benzaldehyde; and m/z 45-acetaldehyde, ethylene oxide. Although these results need to be confirmed, this study presented PTR-MS as a promising method in esophageal cancer screening.^10^

 Regarding the specific evaluation of EB levels of supersulfides, it was revealed that hydrogen disulfide (HSSH), CysSH, CysSSH, and HS_2_O_3_^–^ levels significantly varied between healthy subjects and patients with esophageal cancer, with CysSSH being elevated and HSSH and HS_2_O_3_^–^ being decreased in patients with cancer. As indicated, EB’s CysSSH level was able to distinguish patients with esophageal cancer from healthy individuals with an AUC of 0.71, 60% sensitivity, and 96% specificity, highlighting the role of CysSSH as a potential biomarker for detecting esophageal cancer.^42^

 In addition, a study utilizing the electronic nose device for identifying Barrett’s oesophagus, a recognized precursor to esophageal carcinoma, has yielded promising results. The device showed 91% sensitivity and 74% specificity in differentiating patients with Barrett’s esophagus from healthy controls.^43^

 These findings suggest that EB analysis could be instrumental in early detection of precancerous conditions, facilitating improved monitoring and earlier intervention.

## Hepatocellular Carcinoma

 Hepatocellular carcinoma (HCC) is the most common primary liver cancer, typically developing in the setting of chronic liver disease such as hepatitis B and C infections and cirrhosis. It accounts for approximately 70-90% of primary liver cancers worldwide and is a leading cause of cancer mortality globally.^44^ Regarding the critical role of early-stage diagnosis of HCC in improving treatment options and survival outcomes, a few studies have assessed the use of VOCs as diagnostic biomarkers for HCC. Although the results were remarkable, further evaluation is necessary for applying this method as a non-invasive, widely used diagnostic approach.

 The initial results revealed a total of 18 VOCs with significantly different levels between the case and control groups. By utilizing a combination of six VOCs (acetone, methylene chloride, phenol, benzene, 1,4-pentadiene, and allyl methyl sulfide) a maximum of 79.6% accuracy, 76.5% sensitivity, and 82.7% specificity was achieved, based on the training set. For the test set, the accuracy dropped to 55.4%, with sensitivity at 44.0% and specificity at 75.0%. In terms of early diagnosis for HCC, d-limonene showed 62.8% sensitivity, 51.8% specificity, and 54.9% overall accuracy. Although it demonstrated significantly higher sensitivity, its specificity and accuracy were notably lower when compared to alpha-fetoprotein (AFP). Following treatment, levels of acetone, butane, and dimethyl sulfide were significantly altered. Notably, patients who achieved a complete response exhibited a more marked decrease in acetone levels compared to those with residual tumors after treatment. The reduction in acetone levels effectively indicated treatment response, highlighting the potential of this method for both monitoring and diagnosis.^45^

 A recent study employing the XGBoost algorithm assessed the importance of various VOC features by evaluating their ability to differentiate among three groups: HCC patients, individuals with cirrhosis, and healthy controls. This evaluation resulted in feature importance scores (F scores) for each VOC. The study identified nine VOCs capable of distinguishing between these cohorts, with acetone monomer leading the list, followed by ethanol, acetone dimer, and acetonitrile, all of which achieved the highest F scores. Utilizing these nine VOCs, the study reported 70.0% sensitivity, 88.6% specificity, and 75.0% accuracy for HCC diagnosis.^46^

 The acetone dimer showed promising potential for early HCC diagnosis, effectively distinguishing between cirrhotic patients and those with HCC and achieving a higher AUC than the standard biomarker AFP (0.775 vs. 0.714, respectively). Additionally, acetone dimer showed considerable promise in monitoring the response to HCC treatment. Notably, acetone outperformed AFP in overall effectiveness for HCC diagnosis, while isopropyl alcohol emerged as a potential predictor of survival in HCC patients.^46^

 Furthermore, styrene, 3-hydroxy-2-butanone, and decane were evaluated as potential biomarkers, with 3-hydroxy-2-butanone demonstrating the most promising diagnostic value. The diagnostic function utilizing these markers achieved 86.7% sensitivity and 91.7% specificity in distinguishing between HCC patients and healthy individuals, and yielded 83.3% sensitivity and 91.7% specificity in cross-validation. Notably, this diagnosis was independent of AFP levels or clinical stages.^47^

 Moreover, (E)-2-nonene, ethane, and benzene were identified as the most significantly increased metabolites in HCC patients. However, hydrogen sulfide showed significant reduction in patients with HCC compared to healthy individuals. It is noteworthy that a designed model consisting of 22 metabolites could identify HCC patients with 72% accuracy, 73% sensitivity, and 71% specificity.^48^

## Pancreatic Cancer

 Pancreatic cancer, predominantly pancreatic ductal adenocarcinoma, is marked by genetic and epigenetic alterations that lead to aggressive tumor growth within a dense stromal microenvironment, contributing to therapy resistance. Although less common than other gastrointestinal cancers, it has a high fatality rate due to late diagnosis and limited treatment options.^49,50^ The assessment of VOCs in EBC for diagnosing pancreatic cancer has not been extensively studied. However, given the unfavorable prognosis, the fatal nature of this disease, and its ranking as the fourth leading cause of cancer-related mortality in the United States, finding innovative early diagnostic methods is of significant importance.^51^

 Initially, it was revealed that there were five upregulated VOCs, including acetone, acetoin, formaldehyde, undecane, and isopropyl alcohol, and seven downregulated ones, including pentane, n-hexane, 1-butanol, 1-(methylthio)-propane, benzaldehyde, tetradecane, and amylene hydrate in cancerous samples. ROC curve analysis, utilizing significant VOCs and data from the validation cohort, yielded an area under the curve of 0.736 (81% sensitivity and 58% specificity) for distinguishing cancer from non-cancer cases. Additionally, it achieved an area of 0.744 (70% sensitivity and 74% specificity) for differentiating adenocarcinoma from non-cancer.^52^

 Moreover, a study with a designed predictive model, consisting of 10 VOCs, exhibited a significant and independent association with diagnosing pancreatic ductal adenocarcinoma, achieving 100% sensitivity and 84% specificity, along with an area under the curve of 0.99. In further validation, the model was able to identify 23 out of 24 patients and 25 out of 26 healthy controls.^11^

## Practical Considerations and Limitations

 The utilization of breath analysis for the diagnosis of gastrointestinal malignancies demonstrates considerable promise as a noninvasive strategy, potentially enabling early detection of unique cancer-related markers. Nevertheless, despite its significant potential, numerous practical and methodological barriers persist that prevent its clinical implementation.

 A primary limitation lies in the absence of standardized protocols across study designs. Existing research exhibits significant heterogeneity in patient preparation, as well as in the procedures for collecting, storing, and analyzing breath samples. Such inconsistencies contribute to heterogeneity in study outcomes, which undermines both the reproducibility and reliability of reported findings.^53,54^

 Additionally, numerous confounding variables, including comorbidities, dietary factors, medication, smoking status, and exposure to environmental VOCs, complicate the accurate identification of disease-specific biomarkers. These factors, coupled with the inherent variability of individual breath composition and demographic differences such as age, gender, and ethnicity, obscure the identification of disease-specific biomarkers and emphasize the necessity for population-specific validation.^55^

 Another significant concern is the absence of global reference standards and lack of reproducible multicenter studies; the majority of existing research is limited to small cohorts and single-center settings, which restrict the generalizability of findings. Accordingly, future research should prioritize large-scale prospective trials aimed at establishing robust and clinically applicable biomarker signatures.^56^

 Many breath markers under investigation are not exclusive to specific cancer types, complicating differentiation among distinct gastrointestinal malignancies. Furthermore, distinguishing malignant from premalignant conditions remains challenging due to overlapping VOC profiles, which may lead to false-positive or false-negative results.^57^

 Beyond these biological and methodological issues, the high economic cost of breath analysis presents a significant barrier to its widespread clinical implementation. Predominant analytical platforms, such as mass spectrometry, and electronic nose systems, require costly equipment and highly trained personnel for operation and data interpretation.^56^

 Finally, while breath-based VOC analysis holds considerable potential as a complementary, non-invasive tool for early detection and monitoring of gastrointestinal cancers, significant methodological, biological, and economic challenges must be addressed before it can be used routinely in the clinic.

## Conclusion

 In this review, we highlighted the growing interest in exhaled breath condensate and its components, particularly VOCs, as emerging non-invasive diagnostic tools for gastrointestinal cancers. Current diagnostic methods have notable limitations, including their invasive nature and lack of opportunities for early detection, both critical factors for enhancing patients’ outcomes and prognosis.

 Research findings indicated that several compounds show significant diagnostic promise across multiple gastrointestinal cancer types. In the case of colorectal cancer, several VOCs, including propanal, acetone, benzaldehyde, and an array of hydrocarbons, have demonstrated impressive accuracies. Furthermore, some studies were able to achieve sensitivities exceeding 95% and specificities above 90%. Although preliminary findings are promising, replication in larger cohorts is warranted.

 Gastric cancer research has yielded the same promising results, with biomarkers including 2-propenenitrile, furfural, and 2-butoxy-ethanol consistently appearing across multiple investigations. Reaching diagnostic accuracies of 97% in some studies, combined with the ability to distinguish between early and advanced stages of disease, underscores the great potential for clinical utility of this approach. The correlation between breath VOCs and gut microbiota is also noteworthy and highlights that breath analysis may provide insight into the complex interplay between cancer development and microbial flora.

 Moreover, the application of breath analysis for esophageal, hepatocellular, and pancreatic cancers has also shown considerable promise, with specific VOC profiles enabling discrimination from healthy controls with great sensitivities and specificities. As identified, acetone and its derived components are great biomarkers for hepatocellular carcinoma, which also have the ability to predict treatment response, highlighting the multifaceted applications of this technology beyond initial diagnosis.

 Despite these encouraging findings, several important limitations need to be considered. The variation in VOC profiles between different populations, as mentioned in gastric cancer studies, emphasizes the influence of genetic, dietary, and environmental factors on biomarker expression. This variability underscores the crucial need for development of population-specific reference standards and validation protocols. Additionally, the challenge of distinguishing between malignant and pre-malignant conditions, as observed in colorectal cancer studies with advanced adenomas, indicates the need for enhanced analytical approaches and more sophisticated pattern recognition algorithms.

 Future research should focus on standardizing collection protocols, establishing universal reference standards, and conducting large-scale, multi-center validation studies to confirm the reproducibility and generalizability of these findings. The integration of artificial intelligence and machine learning algorithms, as demonstrated in several studies, will likely enhance diagnostic accuracy and enable the development of more sophisticated predictive models.

 The non-invasive nature, cost-effectiveness, and ease of sample collection in this method make this approach particularly suitable for population-based screening programs, potentially enabling earlier detection of GI cancers in asymptomatic individuals and ultimately reducing the substantial global burden of these diseases.

 In conclusion, exhaled breath analysis represents a transformative advancement in gastrointestinal cancer diagnostics, offering a non-invasive, accurate, and practical alternative to current methods. While challenges remain in standardization and validation, the consistent demonstration of high diagnostic performance across multiple cancer types provides compelling evidence for the continued development and eventual clinical implementation of this innovative technology. The future of gastrointestinal cancer diagnosis may indeed lie in the breath we exhale, fundamentally changing our approach to early detection and patient care.
